# The Effects of Tai Chi Training in Patients with Heart Failure: A Systematic Review and Meta-Analysis

**DOI:** 10.3389/fphys.2017.00989

**Published:** 2017-12-07

**Authors:** Xiaomeng Ren, Yanda Li, Xinyu Yang, Jie Li, Huilong Li, Zhengzhong Yuan, Yikun Sun, Hongcai Shang, Yanwei Xing, Yonghong Gao

**Affiliations:** ^1^Guang'anmen Hospital, Chinese Academy of Chinese Medical Sciences, Beijing, China; ^2^Key Laboratory of Chinese Internal Medicine of Ministry of Education and Beijing, Dongzhimen Hospital Affiliated to Beijing University of Chinese Medicine, Beijing, China; ^3^Department of Surgery, Dongzhimen Hospital Affiliated to Beijing University of Chinese Medicine, Beijing, China; ^4^Department of Traditional Chinese Medicine, First Affiliated Hospital of Wenzhou Medical University, Wenzhou, China

**Keywords:** heart failure, Tai Chi, meta-analysis, 6-min walk distance, left ventricular ejection fraction, B-type natriuretic peptide

## Abstract

Heart Failure (HF) is associated with significantly high morbidity and mortality. We performed a meta-analysis and updated new evidences from randomized controlled trials (RCTs) to determine the effects of Tai Chi (TC) in patients with HF. Electronic literature search of Medline, PubMed, EMBASE, the Cochrane Library, China national knowledge infrastructure (CNKI), and Wan Fang Database was conducted from inception of their establishment until 2017. And we also searched Clinical Trials Registries (https://clinicaltrials.gov/ and www.controlled-trials.com) for on-going studies. A total of 11 trials with 656 patients were available for analysis. The results suggested that TC was associated with an obviously improved 6-min walk distance [6MWD, weighted mean difference (WMD) 65.29 m; 95% CI 32.55–98.04] and quality of life (Qol, WMD −11.52 points; 95% CI −16.5 to −6.98) and left ventricular ejection fraction (LVEF, WMD 9.94%; 95% CI 6.95 to 12.93). TC was shown to reduce serum B-type natriuretic peptide [BNP, standard mean difference (SMD) −1.08 pg/mL; 95% CI −1.91 to −0.26] and heart rate (HR, WMD −2.52 bpm; 95% CI −3.49 to −1.55). In summary, our meta-analysis demonstrated the clinical evidence about TC for HF is inconclusive. TC could improve 6MWD, Qol and LVEF in patients with HF and may reduce BNP and HR. However, there is a lack of evidence to support TC altering other important long-term clinical outcomes so far. Further larger and more sustainable RCTs are urgently needed to investigate the effects of TC.

## Introduction

Heart Failure (HF) is a major public health concern with a serious of clinical symptoms, due to any structural or functional damage of ventricular filling or blood ejection. HF remained a rising global epidemic with an estimated prevalence of over 37.7 million individuals globally (Ziaeian and Fonarow, 2016). In United States, from 2012 to 2014, the morbidity rate of HF increased from 2.2 to 2.5% (Mozaffarian et al., 2015; Benjamin et al., 2017); and from 2005 to 2013, there were 960,000 new HF cases annually (Benjamin et al., 2017). In addition, it was associated with significantly high morbidity and mortality, which remained about 50% within 5 years (Ponikowski et al., 2016). In China, the prevalence rate of HF was approximately 0.9% in 2003 (Zhou et al., 2015). According to this estimate, there were about 4 million cases of heart failure patients in 2015 (Zhou et al., 2015). In 2015, the total costs in United States were 24 billion, and it would be up to 47 billion in 2030 (Benjamin et al., 2017). Over the past few decades, numerous medical-based therapies have been developed for the management of HF (Yao et al., 2010; Kwekkeboom and Bratzke, 2016). Although pharmacologic therapies were often used as the most common methods of symptom palliation, medications were generally not insufficient to completely relieve symptoms, and some medications may be contraindicated in HF (Kwekkeboom and Bratzke, 2016).

Recently, exercise training has become more prevalent in HF therapy. Among those, Tai Chi (TC), also as known as Tai Chi Chuan (TCC), is proverbially practiced in more and more countries as a form of exercise for health. It is a kind of moderate exercise that is based on ancient Chinese martial arts and has gained popularity in western countries as a form of meditative exercise, focusing on coordination of postural and breathing patterns (Wayne and Kaptchuk, 2008; Sun et al., 2014). TC has developed into various kinds of styles during its evolution, including the Chen, Wu, Sun, and Yang styles (Wang et al., 2004). With TC as a popular exercise worldwide, researches are flourishing. Some researchers showed that it has remarkable benefits for physical and mental functions (Wise, 2015). A number of studies (Taylor-Piliae, 2003; Wei and Liu, 2003; Yeh et al., 2004; Barrow et al., 2007; Yeh et al., 2008a,b; Yao et al., 2010; Caminiti et al., 2011; Yeh et al., 2011, 2013; Sun et al., 2014; Sang et al., 2015a,b) for TC in patients with HF have displayed the improvements in symptoms, quality of life (Qol), and exercise tolerance. Therefore, a systematic review and meta-analysis was performed to assess the effects of TC on the patients with HF. Despite a meta article has been previously published (Pan et al., 2013), one of the main limitations of the existed meta-analysis was the small number of included studies. After literatures retrieval, we updated seven new studies. Secondly, we found the new outcomes to assess the effects of TC on the patients with HF, including left ventricular ejection fraction (LVEF), heart rate (HR), and timed get up and go (TUG). In addition, compared with the previous paper (Pan et al., 2013), which lacked the research from China for TC, searched only English databases and included only four studies, we added the Chinese databases in our article.

## Methods

### Search strategy

Published studies were identified and collected through an electronic literature search of Medline, PubMed, EMBASE, and the Cochrane Library from their establishment inception until September 16, 2017. The search strategy terms used were as follows: “tai chi” OR “Tai-ji” OR “Tai Chi” OR “Chi Tai” OR “Tai Ji Quan” OR “Ji Quan, Tai” OR “Quan, Tai Ji” OR “Taijiquan” OR “Taiji” OR “T'ai Chi” and “heart failure” OR “Cardiac Failure” OR “Heart Decompensation” OR “Decompensation Heart” OR “Myocardial Failure” OR “Congestive Heart Failure (CHF)” OR “Heart Failure, Congestive.” In addition, we carried out manual retrieval of the China national knowledge infrastructure (CNKI) and the Wan Fang Database to identify publications. We also searched Clinical Trials Registries (https://clinicaltrials.gov/ and www.controlled-trials.com) for on-going studies. The method of searching study was limited to RCTs with human subjects, which were published in either English or Chinese. And some newly added papers were retrieved by consulting the reference lists of relevant articles.

### Study criteria and selection

Inclusion criteria for studies were applied as follows: (1) Participants: patients were diagnosed with HF; (2) Intervention: any form of Tai Chi, such as “simplified 24 forms,” or use any form of Tai Chi as a part of interventional treatment; (3) Control group: usual care, including pharmacologic therapy, dietary and exercise counseling; only pharmacotherapy; aerobic exercise; endurance training (cycling and walking) or education sessions; (4) Primary outcome measures: 6-min walk distance (6MWD), Qol (applying the Minnesota Living with Heart Failure Questionnaire, MLHF), serum B-type natriuretic peptide or N-terminal pro brain natriuretic peptide (BNP or NT pro-BNP), LVEF, HR; Secondary outcome measures: peak oxygen uptake (peak VO_2_), TUG, Systolic blood pressure (SBP), Diastolic blood pressure (DBP); (5) Study design: RCT was delivered in a complete paper article and was excluded if they were not published in either English or Chinese.

### Data extraction and management

Data were processed in accordance with the Cochrane Handbook for Systematic Reviews of Interventions (Higgins and Deeks, 2008). Data extractions from included studies were retrieved by two reviewers (Yanda Li and Xinyu Yang) following the standardized data extraction forms and collated by a third reviewer (Xiaomeng Ren). Every inconsistency was resolved until a common view was achieved. Primary outcome measures were estimated for 6MWD, Qol, BNP, or NT pro-BNP, LVEF, and HR; Secondary outcome measures included peak VO_2_, TUG, SBP, DBP.

Patient characteristics (e.g., age, sex, HF diagnosis), details of the intervention (including style of TC, duration, frequency, and time), intervention used for the control group and outcomes were also extracted.

### Quality assessment

The risk of bias (Higgins et al., 2011) in included studies was evaluated by the tool with Cochrane Collaboration's recommendation, including domains: random sequence generation (selection bias); allocation concealment (selection bias); blinding of participants and personnel (performance bias); blinding of outcome assessment (detection bias); incomplete outcome data (attrition bias); selective reporting (reporting bias); and other bias. Assessments of the risk of bias were showed in the risk of bias table. The meta-analysis was performed to comply with the Preferred Reporting Items for Systematic Reviews and Meta-Analyses (PRISMA) statement (Schünemann et al., 2011).

### Statistical analysis

For this meta-analysis, all data were analyzed by using Review Manager (RevMan) 5.3.0 software (http://ims.cochrane.org/revman/download) and STATA version 12.0. In addition, ethical approval was not required, because the article is a systematic review and a meta-analysis.

We entered the mean change (post-pre-intervention; Higgins and Deeks, 2008) from the baseline to follow-up and the standard deviations (SDs) of the mean changes of the outcomes for the TC groups and control groups into the RevMan software. To account for this, we extracted the continuous outcomes as the weighted mean difference (WMD). WMD was a historical term also referred to as the mean difference (MD), which was used to measure the absolute difference between the mean values in two groups when measured outcome by the same scale (Higgins et al., 2003; Higgins and Deeks, 2008). Eleven authors provided data for the outcomes of interest, but one study did not report the SDs of the mean, we thus used *t*-values to calculate the SDs (Higgins and Deeks, 2008). One article provided the median change (first quartile, third quartile), so we used the formula to estimate the mean changes and SDs. Heterogeneity in the studies was tested with the *I*^2^ statistic by Higgins (Sterne et al., 2001), studies with an *I*^2^ statistic of 25–50% were took for low heterogeneity, those with an *I*^2^ statistic of 50–75% were took for moderate heterogeneity, and those with an *I*^2^ statistic of >75% were considered for a high degree of heterogeneity. A *P* ≤ 0.1 was considered statistically significant. A fixed-effect model was used in low heterogeneity (*I*^2^ < 75%), a random-effect model was used for higher heterogeneity (*I*^2^ > 75%). To estimate the stability of the studies, we conducted a sensitivity analysis.

Publication bias was detected using the funnel plots with the RevMan software, and asymmetry suggested publication bias. Meanwhile, publication bias was also assessed by Begg's test, Egger's test for the meta-analysis. If the *P*-value was < 0.05, publication bias existed (Sterne et al., 2001).

## Results

### Literature search

**Figure 1:**
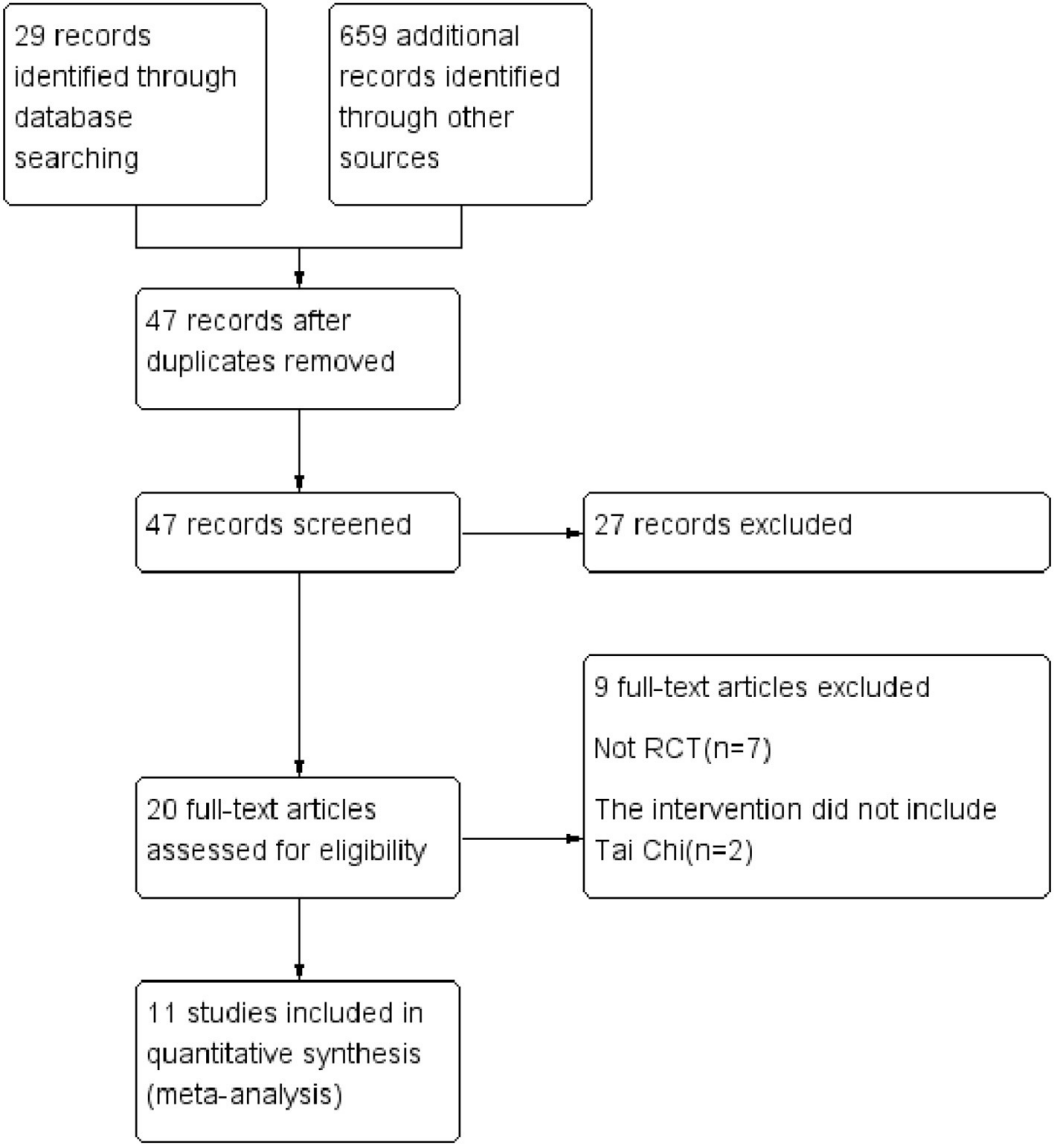
Flow chart for trials included and search strategy for the meta-analysis.

The original qualifying studies from the databases produced 29 candidates and 659 additional records identified through other sources. There were 47 duplicates excluded. According to the study design and after screening study titles and abstracts, 27 records were excluded. Eventually, 11 potentially relevant studies (Wei and Liu, 2003; Yeh et al., 2004; Barrow et al., 2007; Yeh et al., 2008a,b; Yao et al., 2010; Caminiti et al., 2011; Yeh et al., 2011, 2013; Sang et al., 2015a,b) were identified for full-text analysis (Figure 1). Among them, we extracted the same data from two different articles written by one author (Yeh et al., 2004, 2008a). The primary characteristics of these selected reports are described in Table 1. Sample sizes of the included studies ranged from 30 to 150, with a total number of 656 subjects included; 336 were in TC groups and 320 were in control groups (male vs. female, 418 vs. 238, respectively). The mean age was 64.6 years, with a New York Heart Association (NYHA) functional class ranging from I to IV. The duration of TC ranged from 12 to 24 weeks, and exercise time last 15–60 min per session.

**Table 1:** Characteristics of RCTs included in the meta-analysis.

**Author, Year**	**Design**	**Number of patients (M/F), NYHA Class**	**Age (EG/CG), mean; Left ventricular ejection fraction (mean ± SD)**	**Intervention: Tai Chi (TC)**				**Control Group (CG)**	**Outcomes**
				**Style**	**Duration**	**Frequency**	**Time**	**Intervention**	
Yeh et al., 2004, 2008a	RCT	30 (19/11), I–IV	TC Group: 66 years; Control Group: 61 years, 23 ± 7%	Master Cheng Man-Ch'ing's Yang-Style short form Tai Chi and Usual Care	12 weeks	Twice weekly	1 h	Usual care (including pharmacologic therapy and dietary and exercise counseling)	Qol 6-MWD Peak VO_2_ BNP
Barrow et al., 2007	RCT	52 (42/10), II–III	TC Group: 68.4 years; Control Group: 67.9 years, LVEF range not specified	Wu Chian Chuan Style and Chi Kung (including an explanation of the exercise principles involves):derived from the Orchid Hand 21 Style and Wu's Chi Kung exercises	16 weeks	Twice weekly	55 min	Standard medical supervision and drug treatment	SBP DBP Qol
Yeh et al., 2008b^*^	RCT	18 (9/9), I–III	TC Group: 64.2 years; Control Group: 54.7 years, 24% ± 8%	Master Cheng Man-Ch'ing's Yang-Style Tai Chi and performed in cyclic repetition and usual Care	12 weeks	Twice weekly	1 h	Usual care (including pharmacologic therapy and dietary and exercise counseling)	6-MWD Qol
Caminiti et al., 2011	RCT	60 (51/9), II	TC Group: 74.1 years; Control Group: 73.4 years, 33% ± 9%	Yang Style Tai Chi and Endurance Training (Cycling and walking)	12 weeks	3 times Per week	1 h	Endurance training (cycling and walking)	6-MWD SBP DBP NT pro-BNP HR
Yeh et al., 2011	RCT	100 (64/36), I–III	TC Group: 68.1 years; Control Group: 66.6 years, 29% ± 8%	Master Cheng Man-Ch'ing's Yang-Style short form Tai Chi and repetitively	12 weeks	Twice weekly	1 h	Education sessions	Qol 6-MWD Peak VO_2_ BNP TUG
Yeh et al., 2013	RCT	16 (8/8), I–III	TC Group: 68 years; Control Group: 63 years LVEF range not special	Master Cheng Man-Ch'ing's Yang-Style short form Tai Chi and repetitively	12 weeks	Twice weekly	1 h	Aerobic exercise	6-MWD; SBP; DBP; BNP; HR; Qol; TUG; LVEF; Peak VO_2_
Wei and Liu, 2003	RCT	70 (44/26), II–III	60.5, LVEF range not special	Simplified 24 forms	12 weeks	Once weekly	Not mentioned	Pharmacologic therapy	LVEF
Yao et al., 2010	RCT	150 (89/61), II	TC Group: 52.4 years; Control Group: 51.7 ± 7.26 years LVEF range not specified	Chen-style 42 forms	24 weeks	Not mentioned	≥30 min	Usual care (including pharmacologic therapy and dietary and exercise counseling)	LVEF 6-MWD Qol
Sang et al., 2015a	RCT	100 (57/43), II–III	TC Group: 65.3 years; Control Group: 76.2 ± 7.5 years LVEF range not specified	Tai Chi rehabilitation	12 weeks	Once daily	15 min	Pharmacologic therapy	LVEF 6-MWD Qol
Sang et al., 2015b	RCT	60 (35/25), II–III	TC Group: 66.2 years; Control Group: 65.3 ± 6.2 years LVEF range not specified	Tai Chi rehabilitation	12 weeks	Once daily	15 min	Pharmacologic therapy	LVEF BNP

*6-MWD, 6 min walk distance; Qol, quality of life; BNP, serum B-type natriuretic peptide or NT pro-BNP:N-terminal pro brain natriuretic peptide; LVEF, left ventricular ejection fraction; TUG, timed get up and go; Peak VO_2_, peak oxygen uptake; SBP, systolic blood pressure; DBP, diastolic blood pressure; HR, heart rate; TCG, TC Group; CG, Control Group*.

* *We extracted the same data from two different articles written by one author*.

**Figure 2:**
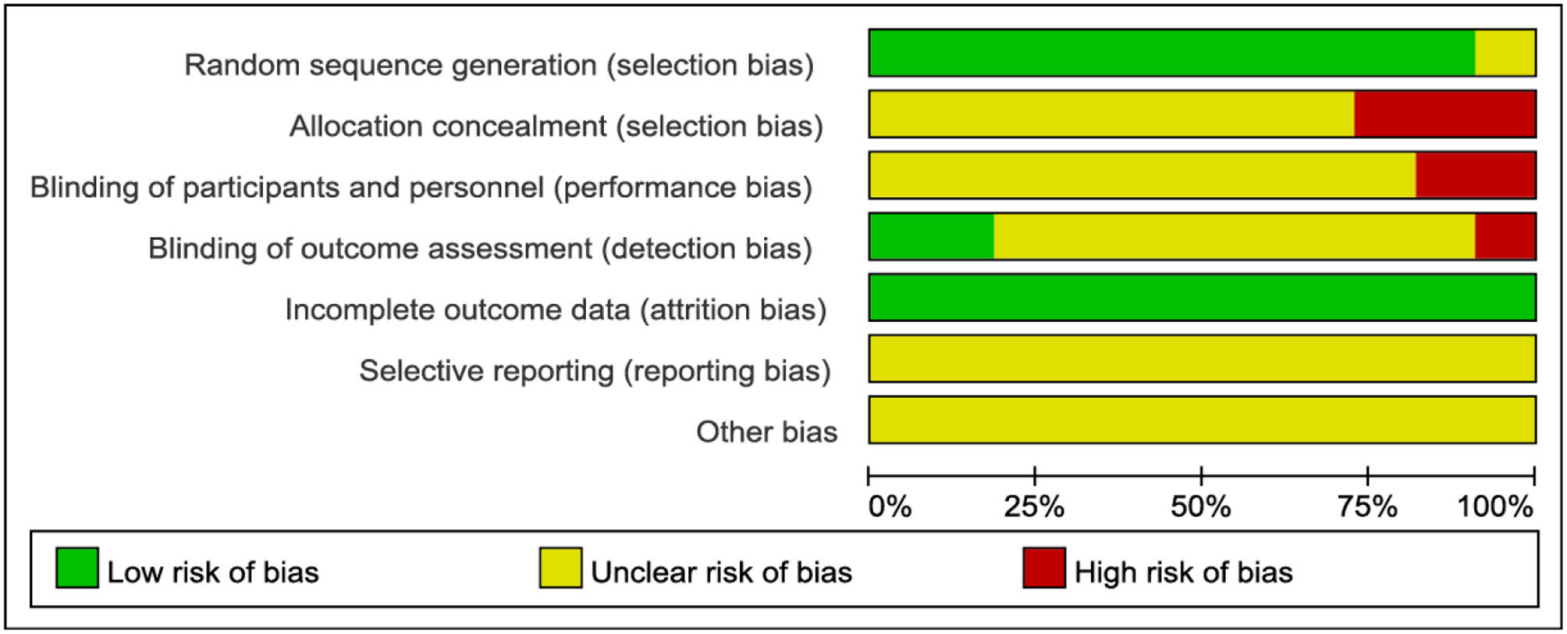
Review judgements regarding each risk of bias item presented as percentages across all included studies.

**Figure 3:**
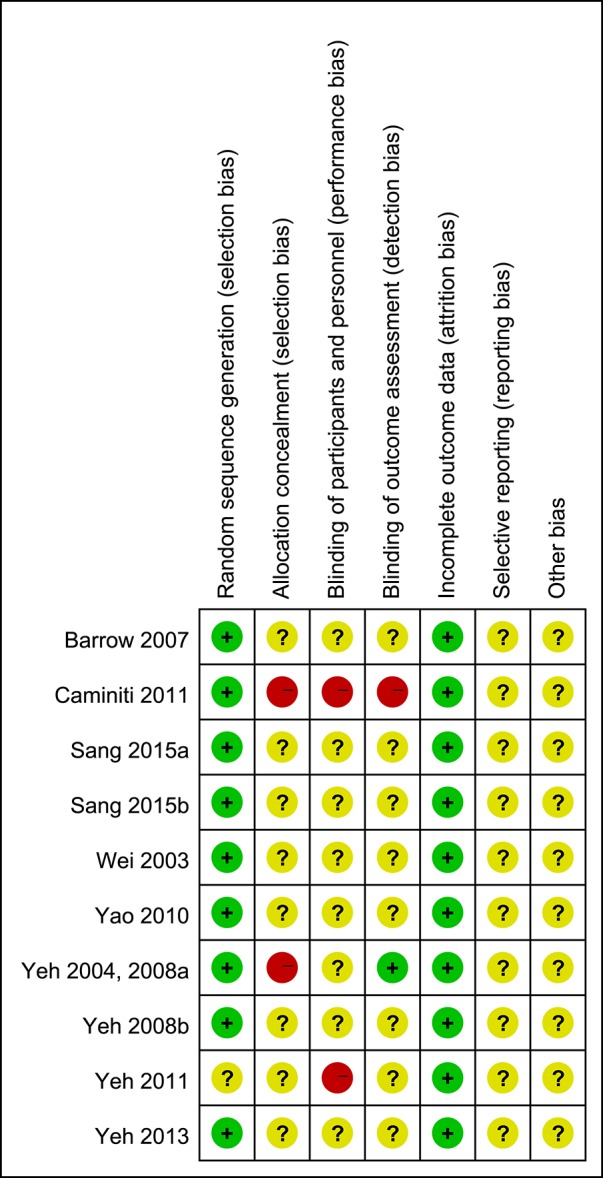
Risk of bias summary: review authors' judgements about each risk of bias item for each included study.

The bias condition of selected studies was shown in Figures 2, 3. We assessed the risk of bias in all included articles. Most articles used the generation of the allocation sequence (*n* = 10, 90.9%). Allocation concealment was inadequate in all articles (*n* = 11, 100%). None of the studies blinded their participants or personnel. And no articles (0%) masked their outcome assessors to the treatment allocation, whereas a risk of selective reporting bias was not reported in all articles (*n* = 11, 100%).

### Primary outcome measures

#### 6MWD (M)

**Figure 4:**
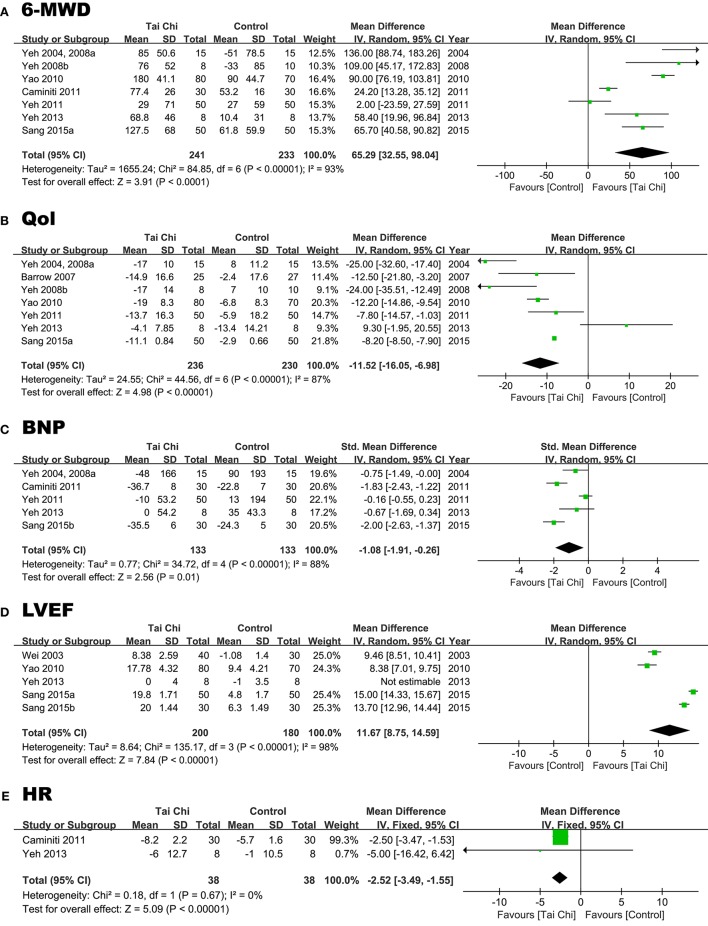
Forest plot comparing changes of the 6MWD **(A)**, Qol **(B)**, BNP **(C)**, LVEF **(D)**, HR **(E)** between the TC groups and the control groups.

Seven RCTs reported this outcome. Combining these seven studies, the results suggested TC was associated with a significantly improved 6MWD (Yeh et al., 2004, 2008a,b; Yao et al., 2010; Caminiti et al., 2011; Yeh et al., 2011, 2013; Sang et al., 2015a). We used the random-effects model and calculated that the WMD was 65.29 m (95% CI 32.55–98.04; *P* < 0.00), which is statistically significant. The test for heterogeneity was also significant (*P* < 0.00, *I*^2^ = 93%; Figure 4A). We excluded individual studies to conduct sensitivity analyses to illustrate the heterogeneity, and the results showed no obvious differences between the selected studies [*I*^2^ = 89% (Caminiti et al., 2011), 94% (Sang et al., 2015a), 87% (Yao et al., 2010), 93% (Yeh et al., 2004, 2008a), 93% (Yeh et al., 2011), 94% (Yeh et al., 2013), 94% (Yeh et al., 2008b)].

#### Qol (point)

Seven RCTs reported the Qol. The results of combining these seven studies indicated obviously lower MLHF scores in the TC group than in control group, which translated to the TC group having a higher Qol (Yeh et al., 2004; Barrow et al., 2007; Yeh et al., 2008a,b; Yao et al., 2010; Yeh et al., 2011, 2013; Sang et al., 2015a). We used the random-effects model and determined that the WMD was −11.52 points (95% CI −16.5 to −6.98; *P* < 0.00), which was statistically significant (Figure 4B). The heterogeneity was calculated as *P* < 0.00 (*I*^2^ = 87%). We did sensitivity analyses to explore potential sources of heterogeneity using the aforementioned method, and the results did not change substantively [*I*^2^ = 89% (Barrow et al., 2007), 84% (Sang et al., 2015a), 80% (Yao et al., 2010), 81% (Yeh et al., 2004, 2008a), 89% (Yeh et al., 2011), 86% (Yeh et al., 2013), 87% (Yeh et al., 2008b)].

#### BNP or NT Pro-BNP (pg/ml)

Because there were two possible outcomes, we referred to both of these parameters as BNP (Yeh et al., 2004, 2008a; Caminiti et al., 2011; Yeh et al., 2011, 2013; Sang et al., 2015b; Figure 4C). Five RCTs reported this outcome. The results of this meta-analysis suggested that TC was correlated with a significantly reduced BNP. Because of the two types of this outcome with different standards, we chose SMD as the result. We used the random-effects model and determined that the SMD was −1.08 (95% CI −1.91 to −0.26; *P* = 0.01), which was statistically significant. The heterogeneity was *P* < 0.000.20 (*I*^2^ = 88%). We excluded any of these five RCTs and the heterogeneity was no pronounced distinction. [*I*^2^ = 87% (Caminiti et al., 2011), 85% (Sang et al., 2015b), 91% (Yeh et al., 2004, 2008a), 70% (Yeh et al., 2011), 91% (Yeh et al., 2013)].

#### LVEF (%)

Five RCTs reported this outcome. The meta-analysis results suggested that TC significantly improved LVEF (Wei and Liu, 2003; Yao et al., 2010; Yeh et al., 2013; Sang et al., 2015a,b). The random-effects model showed that the WMD was 9.94% (95% CI 6.95 to 12.93; *P* < 0.00), which is statistically significant. Additionally, *P* < 0.00 for heterogeneity (*I*^2^ = 98%, Figure 4D). We excluded one of these five RCTs because the heterogeneity was unclear [*I*^2^ = 98% (Sang et al., 2015b), 97% (Sang et al., 2015a), 97% (Wei and Liu, 2003), 98% (Yao et al., 2010), 98% (Yeh et al., 2013)].

#### HR (bpm)

Two RCTs reported the HR (Wei and Liu, 2003; Yeh et al., 2011). The results of combining these studies suggested that TC was associated with a significantly reduced HR. The random-effects model calculated the WMD as −2.52 bpm (95% CI −3.49 to −1.55; *P* < 0.00), which was statistically significant. The heterogeneity was *P* = 0.67 (*I*^2^ = 0%; Figure 4E).

### Secondary outcome measures

#### Peak VO_2_ (mL/kg/min)

**Figure 5:**
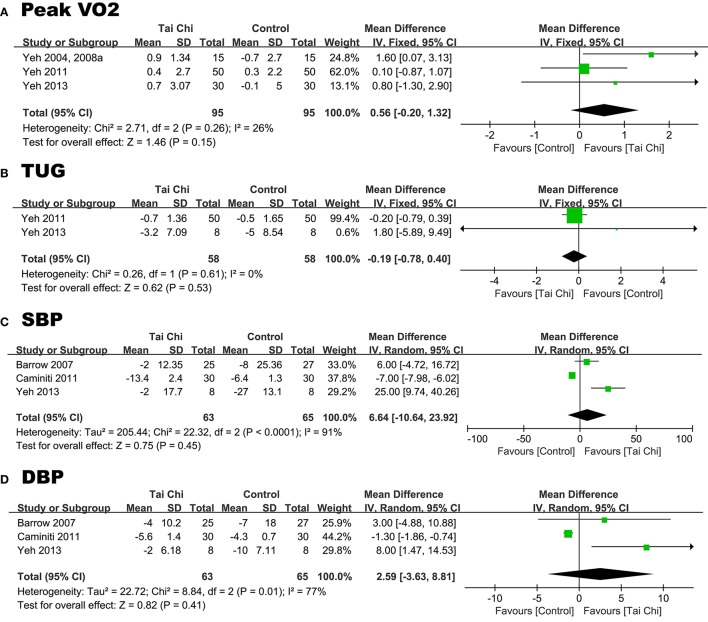
Forest plot comparing changes in the peak VO_2_ **(A)**, TUG **(B)**, SBP **(C)**, DBP **(D)** between the TC groups and the control groups.

Three RCTs reported this outcome. The combination of these results demonstrated that TC did not significantly improve peak VO_2_ (Yeh et al., 2004, 2008a, 2011, 2013). We used the fixed-effects model and determined that the WMD was 0.56 mL/kg/min (95% CI −0.20 to 1.32; *P* = 0.15 > 0.05), which is not statistically significant. The heterogeneity was calculated as *P* = 0.26 (*I*^2^ = 26%, Figure 5A).

#### TUG (s)

Two RCTs reported this outcome. The combined results suggested that TC was not correlated with a significantly decreased TUG (Wei and Liu, 2003; Yeh et al., 2013). We used the fixed-effects model to calculate that the WMD was −0.19 s (95% CI −0.78 to 0.40; *P* = 0.53), which was not significance. The heterogeneity was *P* = 0.61 (*I*^2^ = 0%, Figure 5B).

#### *SBP* (mmHg)

Three RCTs reported the SBP. The meta-analysis revealed that TC did not contribute to any obvious increase of the SBP (Barrow et al., 2007; Caminiti et al., 2011; Yeh et al., 2013). We used the random-effects model to calculate the WMD, which was 6.64 mmHg (95% CI −10.64 to 23.92; *P* = 0.45). The heterogeneity was *P* < 0.00 (*I*^2^ = 91%, Figure 5C). We excluded any RCTs in which *I*^2^ showed no significant change.

#### DBP (mmHg)

Three RCTs reported the DBP. The combined results of these studies indicated that TC was not associated with a significantly improved DBP (Barrow et al., 2007; Caminiti et al., 2011; Yeh et al., 2013). The random-effects model calculated the WMD (2.59 mmHg; 95% CI −3.63 to 8.81; *P* = 0.41 > 0.05), which was not significant. The *P*-value was 0.01 (*I*^2^ = 77%, Figure 5D). Exclusion of the RCTs conducted by Caminiti et al. (2011) and Yeh et al. (2013) altered these results and resolved the heterogeneity [*I*^2^ = 0% (Caminiti et al., 2011), *I*^2^ = 12% (Yeh et al., 2013)].

#### Publication bias and sensitivity analyses

**Figure 6:**
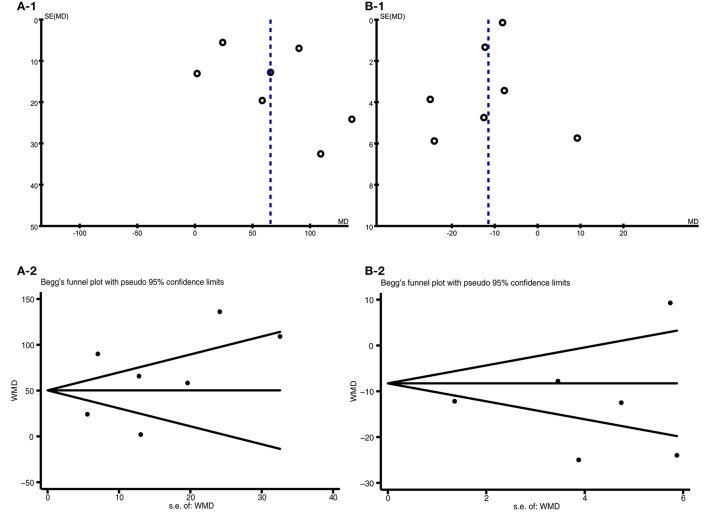
Funnel plot for publication bias. The funnel plot did not show any substantial asymmetry, suggesting no evidence of publication bias. **(A-1)** Funnel plot for the 6MWD outcome as determined using RevMan 5.3; **(A-2)** Begg's publication bias plot of 6MWD; **(B-1)** The funnel plot for the Qol outcome as determined using RevMan 5.3; **(B-2)** Begg's publication bias plot of Qol.

The funnel plots for the Qol and BNP were generated using RevMan 5.3, and the Begg' s funnel plot showed no substantial asymmetry (Figure 6), which suggested no potential publication bias. Egger's regression test of publication bias of the 6MWD (*t* = 0.82 *P* = 0.452) and the Qol (*t* = −1.15 *P* = 0.304) indicated no evidence of publication bias (Table 2).

**Table 2:** Egger's test of publication bias for 6 WMD and Qol.

**Outcome**	**Std_Eff**	**Coef**.	**Std. Err**.	***t***	***P* > *t***	**[95% CI]**
6WMD	Slope	30.16109	28.634	1.05	0.340	−43.44496 103.7671
	Bias	2.326839	2.854068	0.82	0.452	−5.009775 9.663453
Qol.	Slope	−8.027102	0.453078	−17.72	0.000	−9.191776 −6.862428
	Bias	−1.310337	1.143768	−1.15	0.304	−4.250486 1.629812

*Std. Eff., Standard Effect; Coef., coefficient; Std. Err., Standard Error; CI, confidence interval*.

**Figure 7:**
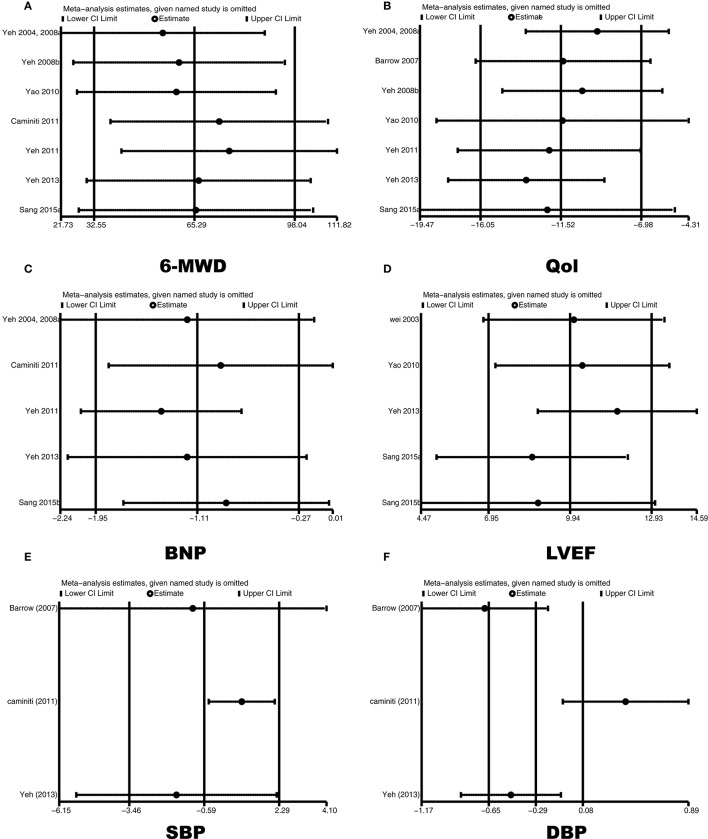
Sensitivity analyses plot of 6MWD **(A)**, Qol **(B)**, BNP **(C)**, LVEF **(D)**, SBP **(E)**, and DBP **(F)** between the TC groups and the control groups.

We excluded each study sequentially, and the remaining studies reporting the DBP had inconsistent results. In the overall meta-analysis, inclusion of the studies by Caminiti et al. (2011) and Yeh et al. (2013) significantly skewed the results, which indicated that these two reports may be statistically unstable (Figure 7F). The other outcomes showed consistent results (Figure 7).

## Discussion

A total of 11 trials with 656 patients were available for analysis. In summary, our meta-analysis demonstrated the clinical evidence about TC for HF. TC may improve 6MWD, Qol, LVEF in patients with HF and could reduce BNP, HR. However, there was a lack of evidence to support TC altering other important long-term clinical outcomes so far, such as recurrence rate, fatality rate, the proportion of aggravated patients.

In this article, our result showed that TC can significantly improve 6MWD compared with the control group. This outcome indicated exercise capacity, and the 6MWD provided information that might be a better indicator compared to peak VO_2_, in terms of the patient's ability to perform daily activities. The 6MWD (Laboratories ATSCoPSf, 2002) was an easy practical test that does a 100-ft hallway, but neither requires exercise equipment nor advanced technical exercise. Walking was a daily activity performed but the most serious patients. This measures the distance that a patient could quickly walk on a flat, hard surface for a period of 6 min. We chose this outcome as a common primary outcome measure; the 6MWD evaluated responses of all the systems involved during exercise, including respiratory system, circulatory system, and motor nervous system. It was more sensitive to deterioration than improvement in HF. TC, as an integrated exercise of the mind-body, which included mind peace, breath flow, body movement, could activate the natural self-healing ability and evoke balanced release of endogenous neurohormones and various natural health recovery mechanisms, so as to improve the heart collateral circulation and increase activity tolerance (Jahnke et al., 2010). Thus, the outcome in our article suggested that TC is sufficient not only to improve functional exercise capacity by focusing on regulating body movement or posture in patients with HF, but also to evaluate the NYHA functional class.

Our findings further demonstrated that TC greatly decreased BNP levels and increased LVEF. BNP was a cardiac neurohormone specifically secreted from the ventricles in response to volume expansion and pressure overload. Biomarkers such as cardiac troponin (cTn) and creatine kinase MB (CKMB) fraction were used to build the diagnosis of acute coronary syndrome and to correlate with both the severity and prognosis of the event; however, there was no gold standard for either the diagnosis or prognoses of CHF in the emergency care setting. The use of the BNP test in conjunction with other clinical evidence should provide more accurate initial diagnoses of CHF (Maisel et al., 2002). In addition, LVEF was one measure that may identify the NYHA functional class. HF was characterized by a series of complex neural and humeral mechanisms; the most important of these mechanisms is the adrenergic nervous system (ANS). This neurohormonal disturbance might play a vital role in HF (Braunwald, 2008). ANS hyperactivity was showed by elevated levels of plasma norepinephrine and epinephrine, increased sympathetic outflow, and plethoric norepinephrine spillover from activated cardiac sympathetic nerve terminals into the circulation, which may lead to the development of left ventricular (LV) diastolic dysfunction, thereby increasing HF risk (Braunwald, 2008). In this meta-analysis, (Yancy et al., 2013) the definition of HF with reduced EF (HFrEF) was varied, with guidelines of LVEF at ≤35, <40, and ≤40%. The included RCTs of patients with HF enrolled HFrEF patients with an EF of either ≤35 or ≤40%, whose NYHA functional class from I to IV; only for these patients, TC therapies have been demonstrated to treat HF through this outcome, whose activity and outflow are enormously elevated in HF. TC, as a moderate intensity exercise, could improve the parasympathetic nervous degree (Lei et al., 2001), inhibit sympathetic activity, increase the coronary collateral circulation, cardiac stroke volume, and cardiac output (Guo and Zhou, 2011), so that it achieved the purpose of reducing BNP to improve LVEF.

TC could obviously slow down HR. ANS could be activated by HF, which exerts various cardiovascular effects such as increasing the HR, increasing cardiac contractility, accelerating cardiac relaxation, and decreasing venous capacitance (Schocken et al., 2008). However, a 10–15% increased risk of HF has been indicated for every increase of 10 beats per minute (Reil and Bohm, 2013). In addition, elevated HR at rest represents a crucial indicator of adverse measure in patients with HF (Lymperopoulos et al., 2013). Angiotensin II (Ang II), which played an significant role in the excessive sympathetic excitation and development of HF, was an important component of the renin–angiotensin–aldosterone system (RAAS) system; its dependence on ANS activation played an vital role in the adverse hemodynamic and LV remodeling responses to myocardial infarction, possibly via oxidative stress (Taylor-Piliae, 2003; Ren et al., 2016). The mechanism of TC exercises may be to maintain the balance of “Yin” and “Yang,” which was a contradiction of unity. Therefore, we should pay attention to the form, spirit and meaning, gas, and other regulations, to lead the body into a state of relaxation; this could be achieved by adjusting the balance of autonomic nerves and reduce the sympathetic nervous tension, thereby adjusting breathing, slowing down the HR and improving the reactivity of the strength and body (Taylor-Piliae, 2003; Ren et al., 2016). Therefore, TC may inhibit ANS, reduce the sympathetic nervous system, and slow down the HR to improve HF.

Another result indicated that the MLHF scores of the TC group were obviously lower than those of the control group, which proved that TC could significantly improve the Qol. Patients with HF often appeared dyspneic and were easily fatigued. Moreover, anxiety, depression, and other negative emotions were often followed by the development of the disease, which contributes to a serious impact on patients' Qol. Thus, Qol could be used as an important outcome for estimating physical health, mental status, and personal beliefs (Lindley et al., 2004), with higher scores indicating worse Qol. TC, which incorporated slow and smooth movements, did not require force; this enables the whole body to relax, so that the cardiac blood supply was enough to not add the rhythm of the heart and increase the burden on the heart. Through slow, long, and uniformed abdominal breathing, the lungs inhale sufficient oxygen, thereby reducing the symptoms of patients, to achieve the purpose of improving the Qol. This outcome therefore rationalized that patients who performed TC regularly may benefit in terms of their Qol. Our findings corroborated the result of another meta-analysis (Pan et al., 2013).

## Limitations

The effects of a TC-training program depended on its style, frequency, and duration. Although published studies have shown that TC was beneficial for patients with HF, this meta-analysis had some potential limitations. First of all, according to the Cochrane Handbook (Higgins et al., 2011), few RCTs performed allocation concealment which was important to reduce heterogeneity (Figures 2, 3). Included studies for TC in this review article presented some problems which led to considerable heterogeneity, such as different testing methods and units of main outcome measures, different control group, HF patients with different NYHA cardiac function classification different style, duration, and frequency of TC training, different TC protocol. Second, the majority of the included studies had a small sample size, and did not undertake the long term follow-up study for TC treating patients with HF. Finally, most of the studies reported positive results, which called into question the possibility of publication bias. The interventions of the control groups in each RCT were inconsistent and different outcome standards were reported, which would generate heterogeneity. Despite these limitations, this review provided a deeply comprehensive analysis of all included studies to express the benefits of TC in patients with HF.

## Conclusion

In conclusion, our results suggested that TC may improve 6-MWD, Qol, LVEF in patients with HF and could be associated with significant reductions in BNP and HR. However, TC did not contribute to any obvious increase of peak VO_2_, SBP, and DBP results. To reduce the TUG was also not obviously significant. Gained more effective data, more targeted and larger well-designed RCTs should be needed to prove current findings and clinical researches the effects of TC on patients with HF. The study in this field is worthwhile and should be continued.

## Author contributions

YG and YX defined the research theme. XR, YL, XY, JL, and ZY designed the methods and analyzed the data. HL, YS, and HS interpreted the results. YX and XR wrote the manuscript. All authors discussed the results and commented on the manuscript.

### Conflict of interest statement

The authors declare that the research was conducted in the absence of any commercial or financial relationships that could be construed as a potential conflict of interest.
